# A Deformed Shape Monitoring Model for Building Structures Based on a 2D Laser Scanner

**DOI:** 10.3390/s130506746

**Published:** 2013-05-21

**Authors:** Se Woon Choi, Bub Ryur Kim, Hong Min Lee, Yousok Kim, Hyo Seon Park

**Affiliations:** Department of Architectural Engineering, Yonsei University, Seoul 120-749, Korea; E-Mails: watercloud@yonsei.ac.kr (S.W.C.); rlaqjqfuf@gmail.com (B.R.K.); hm01.lee@samsung.com (H.M.L.); yskim1220@yonsei.ac.kr (Y.K.)

**Keywords:** displacement sensing, deformed shape, 2D laser scanner, structural health monitoring

## Abstract

High-rise buildings subjected to lateral loads such as wind and earthquake loads must be checked not to exceed the limits on the maximum lateral displacement or the maximum inter-story drift ratios. In this paper, a sensing model for deformed shapes of a building structure in motion is presented. The deformed shape sensing model based on a 2D scanner consists of five modules: (1) module for acquiring coordinate information of a point in a building; (2) module for coordinate transformation and data arrangement for generation of time history of the point; (3) module for smoothing by adjacent averaging technique; (4) module for generation of the displacement history for each story and deformed shape of a building, and (5) module for evaluation of the serviceability of a building. The feasibility of the sensing model based on a 2D laser scanner is tested through free vibration tests of a three-story steel frame structure with a relatively high slenderness ratio of 5.0. Free vibration responses measured from both laser displacement sensors and a 2D laser scanner are compared. In the experimentation, the deformed shapes were obtained from three different methods: the model based on the 2D laser scanner, the direct measurement based on laser displacement sensors, and the numerical method using acceleration data and the displacements from GPS. As a result, it is confirmed that the deformed shape measurement model based on a 2D laser scanner can be a promising alternative for high-rise buildings where installation of laser displacement sensors is impossible.

## 1. Introduction

The structural responses of tall buildings are different in reality from the values calculated from structural analysis at the design stage due to various causes including assumptions in structural modeling for analysis, differences between design drawings and actual work, and changes in service loads. In case of high-rise buildings with a relatively high slenderness ratio greater than 5.0, serviceability of high rise buildings against lateral loads such as wind and earthquake loads must be checked not to exceed the limits on the serviceability-related structural responses. The serviceability-related responses are used for the evaluation of the resistance against deformation and typically include the maximum displacement or deflection, the maximum inter-story drift ratios, and vibration level. Excessive lateral displacement or inter-story drift ratios can cause structural problems as well as other diverse problems on non-structural elements such as damages to finishing materials, while excessive horizontal acceleration level can bring feelings of unpleasantness to building occupants. For these reasons various researches have been conducted on methods of measuring the structural responses of high-rise buildings [1–7].

The responses are mostly monitored by accelerometers, GPS, and image-based monitoring methods. In the case of accelerometers, very precise measurement of acceleration level of a member or structure is possible. However, the lateral displacement must be estimated by numerical integration since displacements cannot be measured directly from an accelerometer [8]. Contrary to the case of accelerometers, GPS allows direct measurement of the displacement history of a high-rise building [1–4]. However, the accuracy of current GPS poses limitations in measurement of lateral displacements since the measured displacement may include about 10 mm errors in the x, y, and z coordinates [9]. The image-based monitoring methods using digital charged coupled device (CCD) cameras have been applied to measurements of structural responses with a relatively high accuracy in the range of several millimeters. The image-based structural behavior monitoring techniques require a number of cameras and installation of a fixed reflective point on target structure. In addition, accurate measurements of the distances between cameras and a fixed point placed outside a target structure are necessary to ensure the accuracy in the measurements. In recent years, a new terrestrial radar interferometry technology [10] has been developed and applied to monitoring of deformations [11,12].

Without any reflective markers or points attached on target structure, three-dimensional coordinate information of target objects can be obtained from terrestrial laser scanning (TLS) [13–15]. As such, application of TLS on the monitoring of the serviceability related responses allows measurements of both three-dimensional displacement and deformed shape of a member in a building [5]. However, there exist many problems to be resolved in measurements by TLS, especially for the serviceability related responses of high-rise buildings in motion, due to the limitation on the sampling frequency in measurements by TLS.

Therefore, in this paper, a sensing model for measurements of the serviceability related responses of a building structure in motion using a 2D laser scanner is presented. The feasibility of the sensing model based on a 2D laser scanner is tested through free vibration tests of a three-story steel frame structure. In the experimentation, the maximum lateral displacement and deformed shapes obtained from the model are compared with those measured directly from laser displacement sensors. In addition, dynamic properties of the three-story steel frame structure obtained both from accelerometers and the proposed model are compared.

## 2. Extraction of Coordinate Data with 2D Laser Scanner

**Figure 1.**
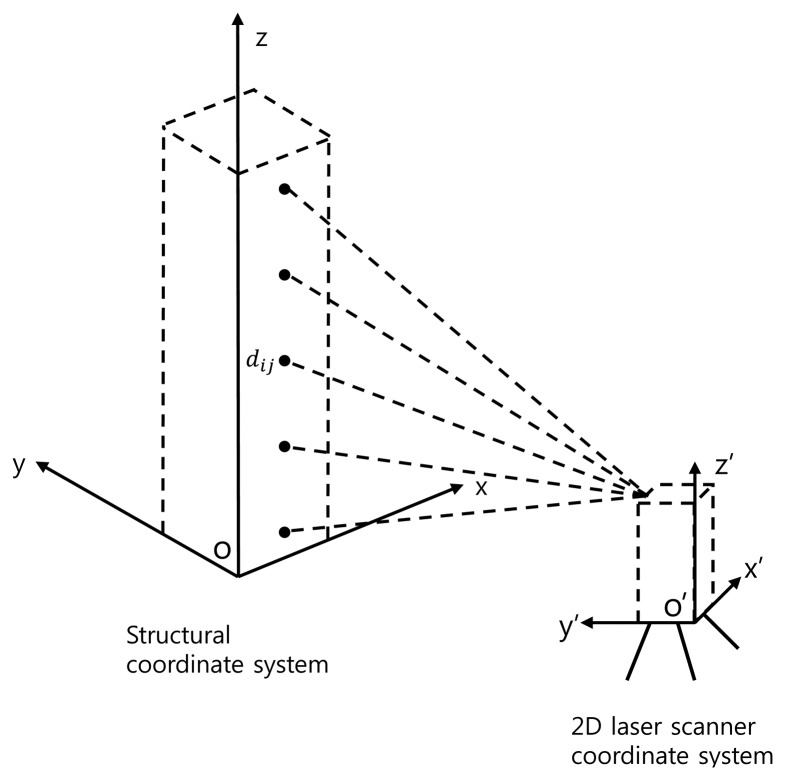
Extraction of 2D coordinate data from the scanner.

A 2D laser scanner uses a laser signal to obtain two-dimensional coordinate data based on the time-of-flight (TOF) principle. The principle of two-dimensional coordinate extraction using a 2D laser scanner involves, as shown in Figure 1, measuring how long it takes for the laser signal to travel and return from its source to an object and computing the distance based on the travel time of the signal. Relative two-dimensional coordinates of an object in reference to the scanner are obtained by using the distance and laser pulse angle data generated by a specific angular interval within the angular range of 80°. Unlike TLS, 2D laser scanners are able to supply a relatively high scanning speed up to 100 scans per second. The scanning frequency of 2D laser scanners makes it possible to measure the deformed shapes of high-rise buildings in motion since the natural frequencies of high-rise buildings with more than 30 stories are less than 1.0 Hz.

## 3. Deformed Shape Measurement Model

To obtain the deformed shape of a building in Figure 1, lateral displacements in the y direction along the z coordinate are measured by a 2D scanner fixed on the ground. Displacements of a point in a structure are computed by comparing the positions of the specific point from the scanner. For the generation of reference points, the structure is scanned once with the fixed scanner. Then, the structure is subsequently scanned for a comparison with the reference points.

Since the coordinate values values *x*'and y' represent the two-dimensional distances from the scanner to each point, the relative coordinate changes between two points in the scanner coordinate cannot be used directly for measurement of lateral displacements in the *x* and *y* coordinate representing the structural coordinate system in Figure 1. For calculation of the relative displacements on the structural coordinate system, a three-dimensional coordinate transformation of including parallel and rotational moves is required. The parallel move constitutes moving the origin $O^{'}(x_{o}^{\prime },y_{o}^{\prime },z_{o}^{\prime })$ of the scanner coordinate system to the origin $O\left(x_{o},y_{o},z_{o}\right)$ of the structural coordinate system, and is expressed by:

(1) $$T_{\text{xyz}}=(-x_{o},-y_{o},-z_{o})$$

**Figure 2.**
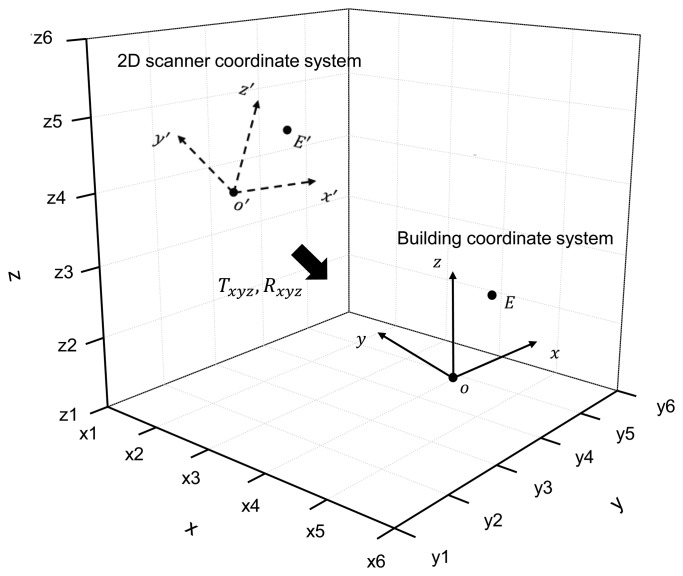
Coordinate transformation from scanner to building coordinates.

The rotational move is transformation of the scanner coordinate system to coincide with the positive direction of axis of the structural coordinate system in Figure 2. By defining the rotational angles *α*, *β*, and *γ* to be transformed along *x*, *y*, and *z* axis, the rotational move is expressed by Equation (2):

(2) $$R_{\text{xyz}}=R_{\alpha }R_{\beta }R_{\gamma }$$

where *α*, *β*, and *γ* are defined by:

(3) $$R_{\alpha }=\left[\begin{array}{ccc} 1 & 0 & 0\\ 0 & \cos\alpha & -\sin\alpha \\ 0 & \sin\alpha & \cos\alpha \end{array}\right]\;,\;R_{\beta }=\left[\begin{array}{ccc} \cos\beta & 0 & \sin\beta \\ 0 & 1 & 0\\ -\sin\beta & 0 & \cos\beta \end{array}\right]\;,\;R_{\gamma }=\left[\begin{array}{ccc} \cos\gamma & -\sin\gamma & 0\\ \sin\gamma & \cos\gamma & 0\\ 0 & 0 & 1 \end{array}\right]$$

To obtain time history of displacement at a specific point in a structure, displacements corresponding to the same point are arranged by Equation (4):

(4) $$d_{ij}=t_{o}+2\Delta_{st}\left(i-1\right)+\frac{\Delta_{st}}{n}j$$

where *d_ij_* is the displacement at the *j*th point (Figure 1) obtained for the *i*th scanning. *t_o_* and Δ*_st_* are the starting time for the scanning process and the time required for one scanning, respectively. *n* is the total number of data points per each scanning.

Two-dimensional coordinates of a point in a building structure obtained from a 2D scanner can have maximum error of about 25 mm. To exploit the advantages of the relatively new technology in field of structural health monitoring, it is necessary to improve the accuracy of 2D scanners. To minimize such errors in coordinate information, in this paper, an adjacent averaging technique is used for smoothing the data from the 2D scanner. In the adjacent averaging, a data point in measured data is replaced by the average of *m* dada points around the point. Then, for a given number *m* of data points to be averaged, the displacement at the *j*th point obtained for the *i*th scanning in Equation (4) is replaced by the average in Equation (5):

(5) $$d_{ij}=\frac{d_{ij-m}+\cdots +d_{ij-1}+d_{ij}+d_{ij+1}+\cdots +d_{ij+m}}{2m+1}$$

**Figure 3.**
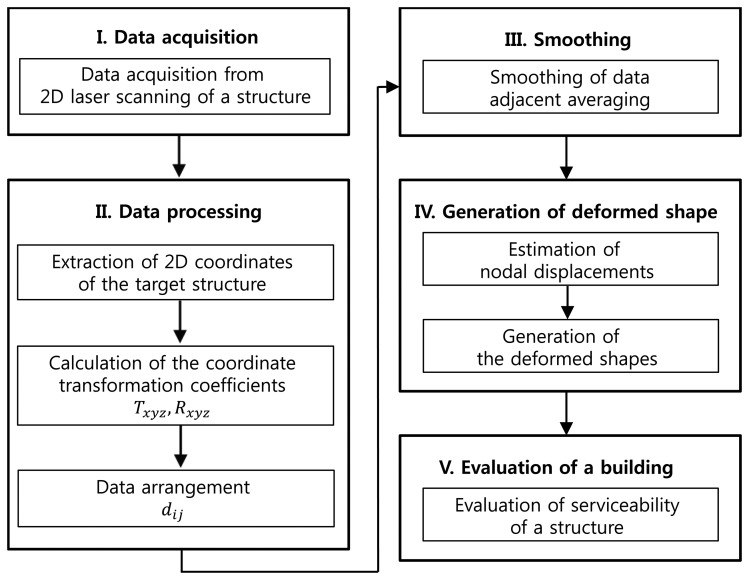
Flowchart for deformed shape monitoring model using a 2D laser scanner.

The flowchart for the deformed shape monitoring model proposed in this paper is shown in Figure 3. As shown in Figure 3, the deformed shape monitoring model based on a 2D scanner consists of the following five modules: (1) module for acquiring coordinate information of a point in a building; (2) module for coordinate transformation and data arrangement for generation of time history of the point; (3) module for smoothing by adjacent averaging technique; (4) module for generation of the displacement history for each story and deformed shape of a building, and (5) module for evaluation of the serviceability of a building.

## 4. Application of the Model

### 4.1. Experimental Setup

**Figure 4.**
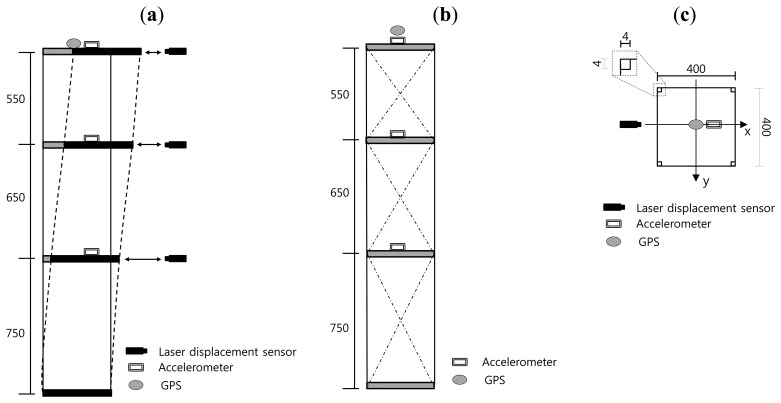
Experimental setup. (**a**) Front view; (**b**) Side view; (**c**) Plan view.

A schematic of the test model is shown in Figure 4. The test model is a three-story three-dimensional steel moment frame with one span of 400 mm in both the x and y directions. The story heights for the 1st, 2nd and 3rd floors are 750, 650 and 550 mm, respectively, therefore the slenderness ratio of the model is 2,000/40 = 5.0, which is similar to the slenderness ratio of high-rise buildings. All columns have the same steel bar section of 4 × 4 mm shown in Figure 4(c). The material for the columns of the test frame is SS400 grade steel with a modulus of elasticity of 206 GPa and yield strength of 235.3 MPa.

The model was set into free vibration along x-direction with a given initial displacement. As shown in Figure 4(a), in this experimentation, the initial displacement of free vibration was set to 50 mm in x-direction at the top of the model. In order to eliminate y-direction oscillation component during free vibration, diagonal bracing members in Figure 4(b) have been installed.

### 4.2. Measurements

**Figure 5.**
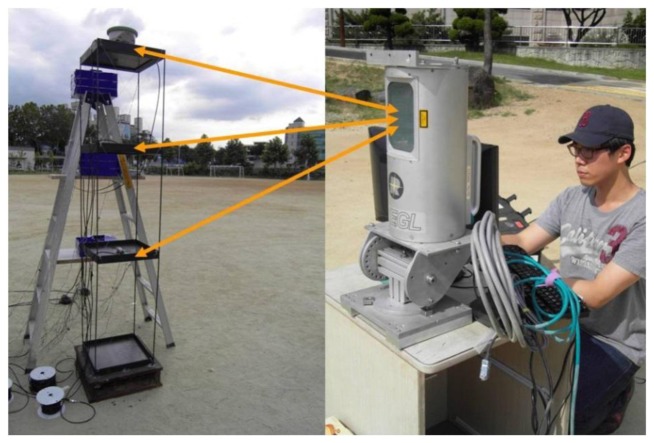
An overall view of experimental setup.

To test the performance of the proposed model, the lateral displacements at each floor level during the free vibration were measured by laser displacement sensors, a 2D laser scanner, and a GPS receiver. As the reference for comparison with the model, actual lateral displacements were measured by three laser displacement sensors [16] on every floor level as shown in Figure 4(a,c). To obtain deformed shapes from the model, lateral displacements at three different floor levels were obtained from the 2D laser scanner located at 4 m from the test model, as shown in Figure 5.

**Figure 6.**
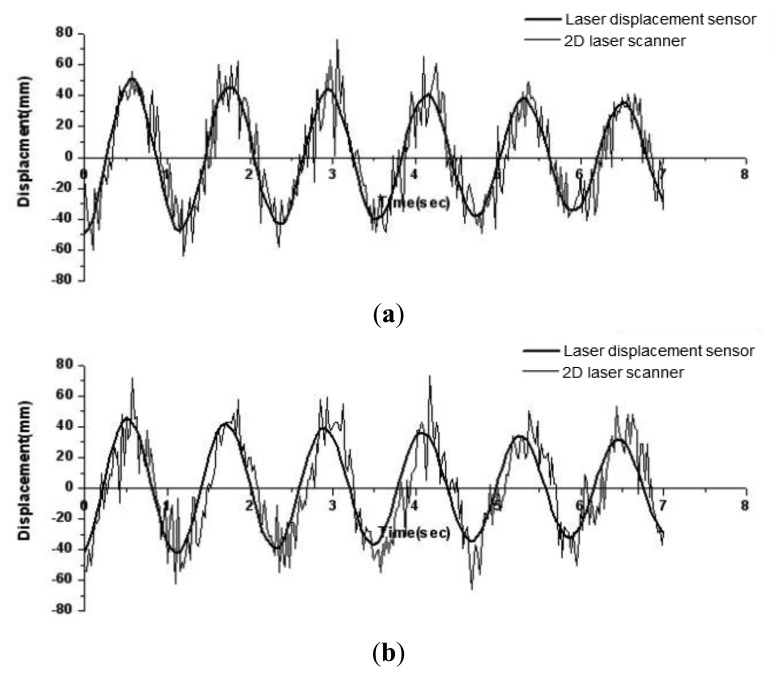

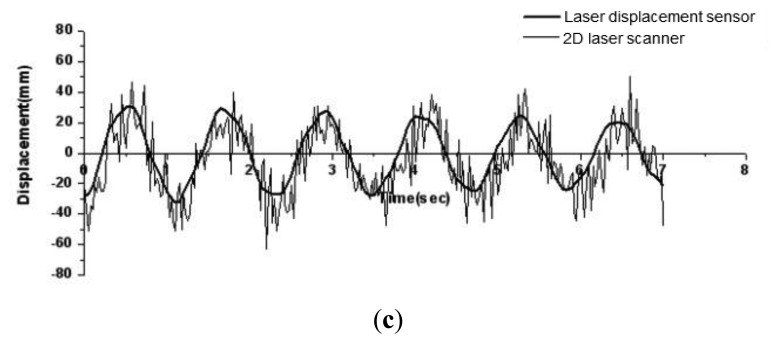
Displacement histories measured by laser displacement sensors and the 2D laser scanner. (**a**) 3rd floor; (**b**) 2nd floor; (**c**) 1st floor.

In this test, *RIEGL* LMS-Q120 2D laser scanner [17] is fixed at the ground to obtain two-dimensional coordinate data of every floor level. The scanner has a divergence of 2.7 mrad corresponding to 27 cm increment of beam width per 100 m of range. During the measurement, the scan angle range was set to be 80° and 200 data points were obtained per each scan. It was assumed that there is no relative lateral displacement among data points in the slab at the same floor. With the initial displacement of 50 mm at the top of the test model, the free vibration responses of the test model measured from both laser displacement sensors and 2D laser scanner are compared in Figure 6.

It is shown that the periods of the free vibration responses at every floor level were almost identical, but the errors in amplitudes become larger for lower stories where the amplitudes in responses are relatively small. From the fast Fourier transform (FFT) analysis of the lateral displacement histories at the 3rd floor, the periods of the first mode for the displacement histories from laser displacement sensors and the 2D laser scanner were found to be 1.18 s and 1.17 s, respectively. However, due to the noise in measurement from the 2D laser scanner, the natural frequencies or periods for 2nd and 3rd modes can't be identified. The averages of errors in maximum displacements in six consecutive cycles in Figure 6 for 3rd, 2nd, and 1st floor levels are about 4.4%, 10.2%, and 22.3%, respectively. It is notable that the maximum amplitude of displacements at the 1st floor lever is about 30 mm, which is similar with the vendor quoted accuracies of about 25 mm errors in range.

**Figure 7.**
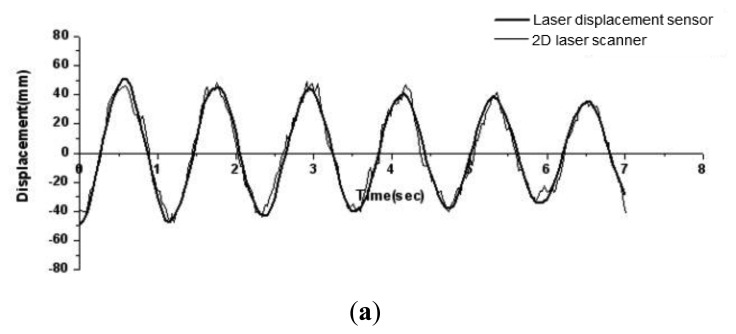

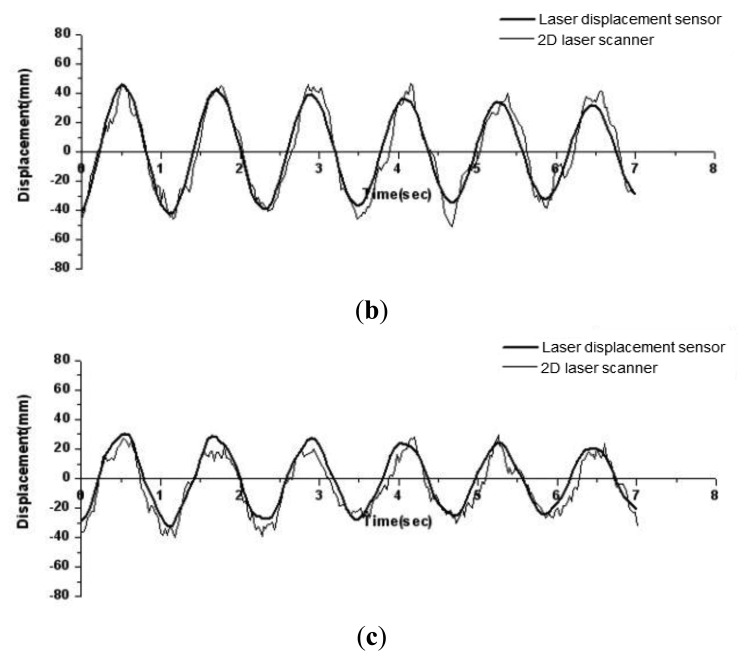
Comparison of the smoothed time history responses with directly measured responses from laser displacement sensors. (**a**) 3rd floor; (**b**) 2nd floor; (**c**) 1st floor.

To smooth the responses obtained from 2D scanner in Figure 6, the adjacent averaging technique with in Equation (5) is applied. After the smoothing, the smoothed time history responses are compared with those directly measured from laser displacement sensors in Figure 7. The average of errors in maximum displacements in six consecutive cycles for 3rd floor level in Figure 7(a) is reduced to less than about 3.2%. However, for both 2nd and 1st floor levels, the average of errors in maximum displacements in six consecutive cycles is about 5.2%.

In general, as one of the representative indices of serviceability design, the maximum lateral displacement at the top of a high-rise building is checked not to exceed a specified limit, which is in the range of 1/400–1/600 of the building height [18,19]. In the case of a high-rise building with the height of 200 m, the limit for the maximum lateral displacement is given in the range of 333.33 mm to 500 mm. Taking the range of displacement limits for high-rise buildings into consideration, it may be worth considering application of the proposed model in monitoring of deformed shapes for high-rise buildings.

### 4.3. Deformed Shapes

**Figure 8.**
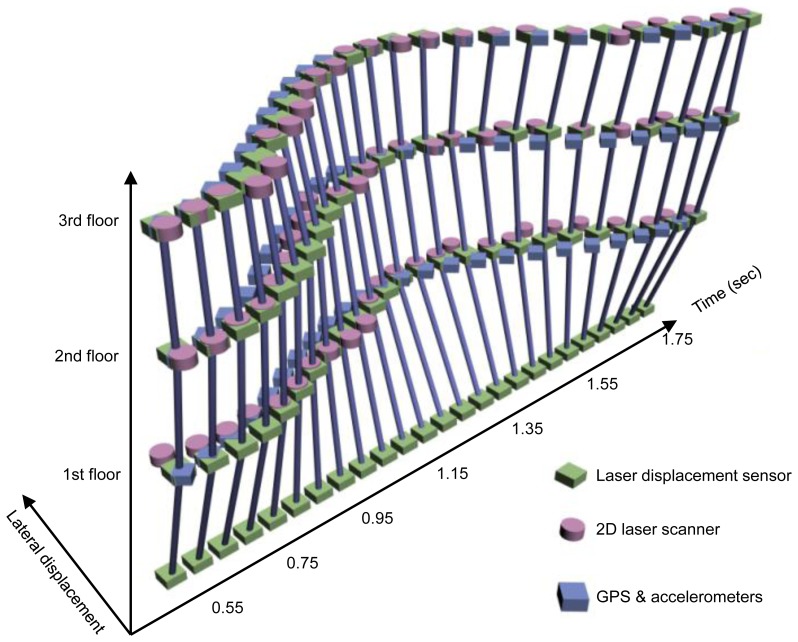
Comparison of deformed shapes of the test model.

Deformed shapes of the test model in Figure 4 during the free vibration can be obtained using the smoothed lateral displacement histories at every story level in Figure 7. Deformed shapes for a one cycle of the vibration from 0.55 s to 1.75 s in Figure 7 are visualized and compared in Figure 8. As can be seen in Figure 8, the difference in magnitudes of lateral displacements at 2nd and 1st story levels are greater than those in 3rd floor level.

**Figure 9.**
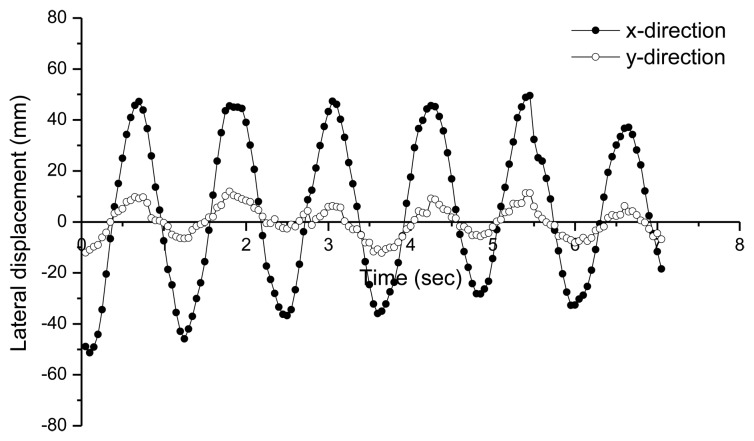
Displacement histories in both x- and y-directions measured by GPS.

**Figure 10.**
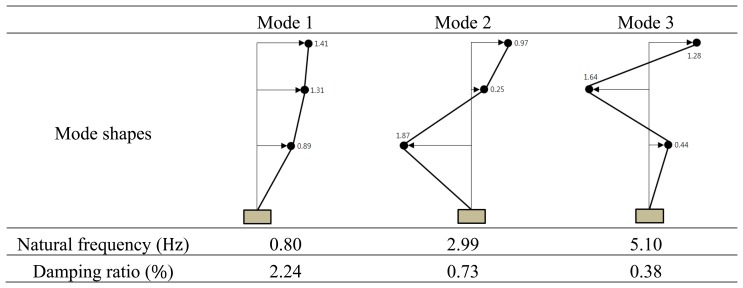
Modal parameters obtained from SSI method.

For comparison of deformed shapes, a GPS receiver is installed at the top of the test model in Figure 5 and three accelerometers [20] (Figure 4(a)) are installed at every floor level. Displacement histories in both x- and y-directions measured by GPS are shown in Figure 9. As a numerical estimation of the deformed shape using stochastic subspace identification (SSI) method [21], the maximum displacement from the GPS receiver at the top of the model can be used to generate deformed shapes based one the mode shapes obtained from SSI method. The SSI method is a numerical tool for finding modal parameters including mode shapes of a structural system using vibration data measured from accelerometers. Based on accelerations measured by three accelerometers, natural frequencies and damping ratios obtained from SSI method are summarized in Figure 10.

The maximum displacement of 49 mm measured from the GPS receiver on the top of the test model is used for calculation of deformed shapes. Deformed shapes based on the modal parameters in Figure 10 and the maximum displacement of 49 mm from the GPS receiver are compared in Figure 8. Due to the error between the initial displacement of 50 mm and the displacement of 49 mm from GPS, besides numerical errors in SSI method, differences in magnitudes of lateral displacement at every story level are easily found.

**Figure 11.**
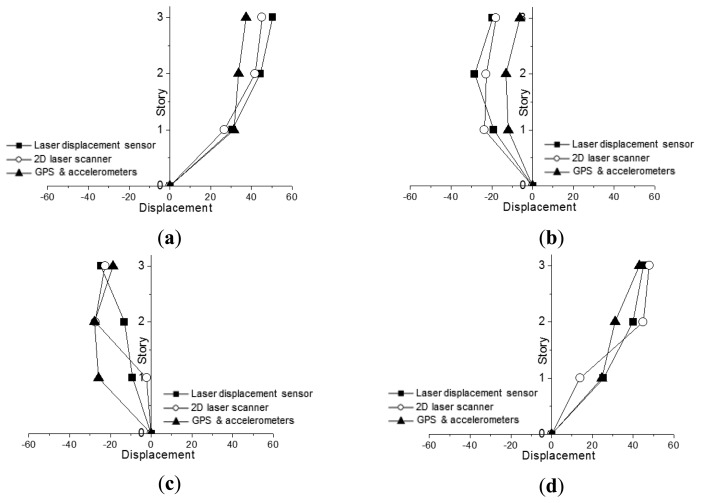
Deformed shapes from three different methods at a discrete time. (**a**) 0.55 second; (**b**) 0.95 second; (**c**) 1.35 second; (**d**) 1.75 second.

For clear comparison of deformed shapes obtained from three different methods, Figure 11 shows deformed shapes at discrete time interval of 0.2 s for the cycle (from 0.55 s to 1.75 s) in Figure 8. It displays that the deformed shapes based on 2D laser scanner are slightly better than those from the numerical method with GPS. The differences in magnitudes of lateral displacements at 2nd and 1st floor levels are relatively greater than those at 3rd floor level. Even though there are many issues to be considered for improvement in precision of measurement, the deformed shape measurement model based on a 2D laser scanner can be a promising alternative for high-rise buildings where installation of laser displacement sensors is impossible.

## 5. Conclusions

In case of a high-rise building subjected to lateral loads such as wind and earthquake loads, monitoring of deformed shapes is necessary to check the serviceability of the building since excessive lateral displacement or inter-story drift ratios can cause structural problems as well as other diverse problems on non-structural elements. In this paper, a sensing model for measurements of the serviceability related responses of a building structure in motion using a 2D laser scanner is presented. Due to the fact that 2D laser scanning provides independent measurements in short times and engineers doesn't need to access the structure, the deformed shape sensing model based on a 2D laser scanner can measure deformed shapes from a safe working distance. To test the performance of the proposed model, deformed shapes of the experimental model in free vibration were obtained and compared to deformed shapes from two different methods.

It was shown that the periods of the free vibration responses at every floor level were almost identical, but the averages of errors in maximum displacements in six consecutive cycles for the 3rd, 2nd, and 1st floor levels are about 4.4%, 10.2%, and 22.3%, respectively. After the smoothing the average of errors in maximum displacements in six consecutive cycles for 3rd floor level was reduced to less than about 3.2%. However, for both the 2nd and 1st floor levels, the average of errors in maximum displacements in six consecutive cycles was about 5.2%. Taking the range of displacement range of high-rise buildings into consideration, it may be worth considering application of the proposed model in monitoring of deformed shapes for high-rise buildings. From the comparison of deformed shapes obtained from three different methods, even though there are many issues to be considered for improvement in the measuring distance and the precision of measurements, the deformed shape measurement model based on a 2D laser scanner can be a promising alternative for high-rise buildings where installation of laser displacement sensors is not possible.
